# Dermatosis Associated with Feeding Low-Quality Food (Generic Food Dermatosis): A Case Series

**DOI:** 10.3390/vetsci13010106

**Published:** 2026-01-21

**Authors:** Alejandro Blanco, Melisa López, Laura Kantor, Adriana Duchene, Lluís Ferrer

**Affiliations:** 1 Servicio de Dermatología del Hospital Escuela, Facultad de Ciencias Veterinarias, Universidad de Buenos Aires, Ciudad Autónoma de Buenos Aires C1427CWO, Argentina; lopezderm@gmail.com; 2 Independent Researcher, Ciudad Autónoma de Buenos Aires C1427CWO, Argentina; laurakantormv@hotmail.com; 3 Servicio de Patología del Hospital Escuela, Facultad de Ciencias Veterinarias, Universidad de Buenos Aires, Ciudad Autónoma de Buenos Aires C1427CWO, Argentina; duchene@fvet.uba.ar; 4 Departament de Medicina i Cirurgia Animals, Facultat de Veterinària, Universitat Autònoma de Barcelona, Bellaterra, 08193 Barcelona, Spain; lluis.ferrer@uab.cat

**Keywords:** generic food dermatosis, nutritional dermatosis, zinc-deficiency dermatosis

## Abstract

Severe crusting dermatosis affecting the mucocutaneous junctions, pressure points, and trunk of dogs fed low-quality dry food was first reported in the United States in the 1980s. Since then, only a few cases have been documented. Twenty-two adult dogs owned by private individuals were evaluated. All dogs exhibited thick crusts forming plaques and marked scaling on the face—particularly around the lips, nasal bridge, and eyelids—as well as on the paw pads, dorsal digits, abdomen, and dorsum. Pruritus ranged from moderate to severe, and all dogs showed varying degrees of lethargy and reduced activity. Each dog was fed a low-quality commercial diet. A presumptive diagnosis of dermatosis associated with poor-quality food was made. Skin biopsies from nine dogs revealed similar histopathologic changes, characterized by epidermal hyperplasia, severe parakeratotic hyperkeratosis with spongiosis, and exocytosis of lymphocytes and neutrophils. The skin lesions resolved within 15–30 days after switching to a high-quality diet. This case series highlights that dermatosis associated with low-quality food should remain in the differential diagnosis for dogs presenting with symmetric crusted and scaly lesions on the face, mucocutaneous junctions, and paws, particularly when nutritional deficiencies are suspected.

## Simple Summary

This study describes 22 dogs that developed severe dermatosis characterized by thick crusts, scaling, and symmetrical lesions on the face, mucocutaneous areas, and paws. All affected animals were exclusively fed low-quality commercial dog food. Histopathology in nine cases showed changes compatible with nutritional deficiencies, particularly marked parakeratotic hyperkeratosis and spongiosis. Other common causes of similar skin lesions, such as infections or autoimmune diseases, were ruled out. The aim of this work was to document this condition—similar to the “generic food dermatosis” first reported in the 1980s—and to demonstrate that it still occurs in clinical practice. After switching to a high-quality, nutritionally complete diet, all dogs improved rapidly: lethargy resolved within one week, and skin lesions healed within 15 to 30 days. This study shows that dermatosis associated with poor-quality diets remains present in some regions and should be considered in dogs with crusted, scaly lesions, especially when they are eating inexpensive, low-quality food. Awareness of this disease supports quicker diagnosis, prevents unnecessary treatments, and highlights the societal importance of access to balanced and nutritious diets for animal health.

## 1. Introduction

The skin is a complex organ with multiple essential functions, including protecting the organism from external damage, maintaining body temperature and water balance, and transmitting sensory information. These functions largely depend on the composition of nutrients within the host’s skin ecological environment. Being a metabolically active organ, the skin has high demands for energy, protein, and various essential nutrients, including vitamins and minerals [1]. Unhealthy diets can lead to a range of skin disorders, affect the skin’s condition from youth to aging, and cause skin injury. In contrast, a balanced diet is crucial not only for the restoration of damaged skin but also for maintaining the skin’s phenotype and homeostasis [2,3].

Deficiencies in essential amino acids, fatty acids, vitamins, and minerals can lead to structural and functional alterations of the skin. Epidermal atrophy may develop in cases of protein, caloric, or vitamin deficiencies. In particular, deficiencies in magnesium, zinc, pantothenic acid, pyridoxine, biotin, vitamin A, or essential fatty acids can result in dermatological disorders characterized histologically by hyperkeratosis, parakeratosis, and acanthosis. Such alterations have been described in several diseases, including canine distemper, pemphigus foliaceus, discoid lupus erythematosus, zinc-responsive dermatosis, ichthyosis, and superficial necrolytic dermatitis [3,4].

The condition known as generic food dermatosis was first described by Sousa and colleagues in 1983 [5] and later expanded in 1988 with a detailed report of 13 affected dogs in the United States between 1982 and 1983 [6]. The dogs had been fed corn- and wheat-based commercial dry diets that failed to meet the nutritional recommendations established by the National Research Council (NRC) for balanced canine maintenance. After two to four weeks of consuming these diets, the animals developed a characteristic clinical picture consisting of lethargy, fever, generalized lymphadenopathy, and a severe crusting dermatosis predominantly affecting the mucocutaneous junctions, pressure points, and trunk. Histopathological evaluation revealed epidermal hyperplasia, parakeratosis, and spongiosis consistent with nutritional deficiency dermatoses. Remarkably, all dogs experienced complete clinical recovery following dietary correction with balanced food meeting NRC standards.

In 1989, Thoday [7] referred to this syndrome—termed diet-related skin disease responsive to zinc—as a “dying disease,” emphasizing that its occurrence appeared to be declining, likely as a consequence of improved quality control and formulation of commercial pet foods. Indeed, the introduction of stricter manufacturing guidelines and greater awareness of canine nutritional requirements during the late 1980s and 1990s substantially reduced the incidence of diet-associated dermatoses.

Since the seminal reports by Sousa and coworkers [6], only two additional cases have been documented in the scientific literature, suggesting that the condition has become exceedingly rare. These involved two six-month-old littermates from Ecuador that presented with similar dermatological lesions and poor body condition while consuming low-quality food [8]. Both animals demonstrated a marked and rapid improvement after being transitioned to a nutritionally balanced diet. Despite these sporadic reports, nutritional dermatoses remain of clinical interest, particularly in regions where access to high-quality commercial foods is limited or where homemade or generic diets are still used. Re-evaluating this entity may provide valuable insight into the dermatological manifestations of dietary deficiencies and reinforce the importance of adequate nutritional standards in canine diets.

In this context, we describe a series of dogs that developed a dermatological disease with remarkable clinical and histopathological similarity to that described by Sousa in 1988, directly related to the consumption of a low-quality diet. The main objective of this study is to demonstrate that, although this entity has progressively disappeared from many contemporary veterinary textbooks and journals, the disease still occurs and remains a reality in certain geographical regions.

## 2. Materials and Methods

Twenty-two dogs from private owners who came to our practices referred by colleagues were included in the study. Each owner gave his/her consent for the study. Breeds, sex, and ages of the 22 patients can be found in Table S1. All dogs were fed low-quality commercial food, which was cheaper than the premium line foods found in the country, and which was marketed through non-veterinary channels. As mentioned, it was a low-priced commercial food, whose composition was indicated on the packaging and is listed in Table 1.

Ingredients: corn, wheat, rice, beef meal, corn gluten, wheat bran, soybean meal, animal fat (beef, pork and/or chicken), sunflower oil, oats, chicken meal, fish meal, flax, chicken protein hydrolysate, brewer’s yeast, salt, zeolite, calcium carbonate, potassium chloride, beet pulp, Yucca shidigera extract, fructooligosaccharides, glucosamine, BHT, BHA, caramel color, calcium propionate, iron (as ferrous sulphate), manganese (as manganese oxide), copper (as copper sulfate), zinc (as zinc oxide), iodine (as potassium iodate), selenium (as sodium selenite), vitamins A, D3, E, K, B1, B2, B6, B12, folic acid, pantothenic acid, niacin, biotin, choline.

Six dogs (27%) had been consuming this food for more than six months and 16 (72%) for less than 6 months.

### 2.1. Clinical Examination

**Figure 1:**
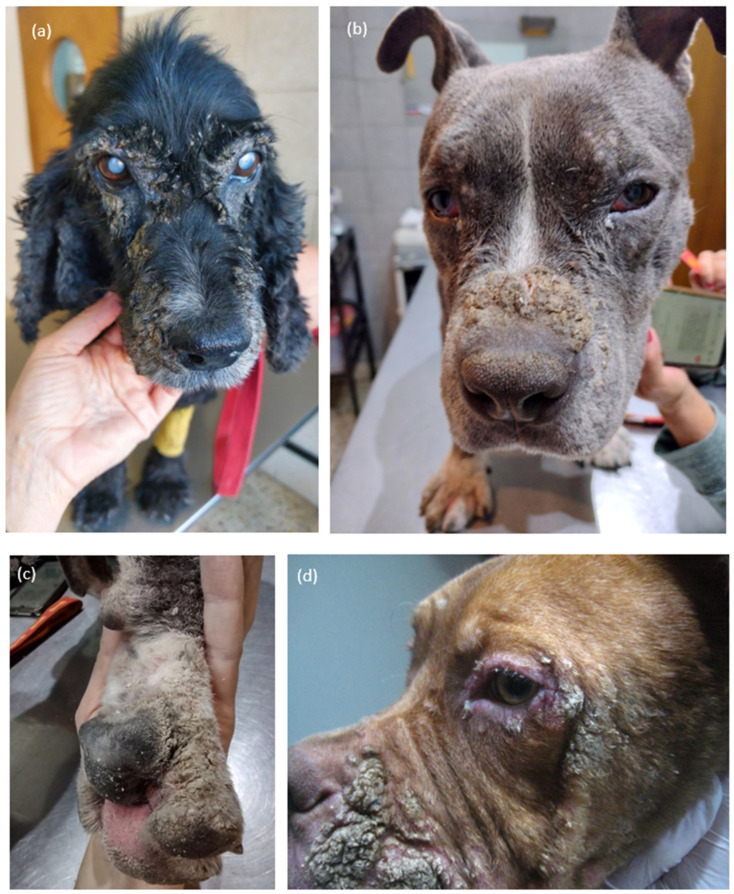
(**a**) Case 15. Seven-year-old, spayed female cocker spaniel, showing severe crusting around the eyes and on the lips. (**b**,**c**) Case 4. Three years old, castrated, male pit bull showing severe crusting on the dorsal nose and generalized scaling on the head and the paw pads. (**d**) Case 9. One-year-old, intact male pit bull. Severe crusting on the paws, including pads and interdigital skin.

The dermatological examination revealed a uniform pattern characterized by thick crusts, forming plaques, and severe scaling, which were symmetrically distributed on the face (Figure 1a), mainly around the lips, bridge of the nose, and eyelids and periocular skin (Figure 1b,d), but also on the paw pads (Figure 1c), dorsum of the toes, abdomen, and dorsum. The process was accompanied by pruritus varying from moderate to severe. All dogs presented some degree of lethargy and reduced activity.

### 2.2. Laboratory Tests

Skin scrapings, microscopic examination of plucked hairs, and Wood’s lamp examination (Gen^®^ 2020 Medicatech, Buenos Aires, Argentina) were carried out. Cytology of the skin surface was performed with a Diff-Quik stain and revealed desquamate keratinocytes and signs of superficial pyoderma with inflammatory cells and intracellular cocci in 4 of the 22 dogs.

It was not recorded whether the patients had complete internal or external deworming, but no ectoparasites were seen in any patient at the time of the consultation. None of the patients had a history of having suffered from any previous dermatological pathology.

Seven dogs (32%) underwent hematology and serum biochemistry, with no significant abnormalities detected (Table 2) Five of those patients subsequently underwent biopsies. Two patients underwent abdominal ultrasonography, which revealed only a hypoechogenic liver image with fine echogenicity and no alteration of the vasculature, regular borders and normal size.

### 2.3. Histologic Examination

Skin biopsies were obtained from 9 dogs (40%). Three samples were taken from each of the 9 patients. The samples were routinely processed and stained with H/E and PAS for detection of possible dermatophytes.

## 3. Results

A presumptive diagnosis of dermatosis associated with low-quality food was established in all 22 dogs based on clinical history and characteristic dermatological findings. The dogs exhibited marked crusting, severe scaling, and erosions primarily on the face—especially around the lips, nasal bridge, and eyelids—as well as on the plantar pads, dorsal aspects of the toes, abdomen, and dorsal-lumbar region. These lesions were generally symmetric and associated with variable degrees of pruritus, erythema, and secondary bacterial colonization in some cases. The skin scrapings were all negative, as was the observation with the Wood’s lamp (Gen^®^ 2020). Cytology tests showed bacterial infection in 4 of the 22 patients.

**Figure 2:**
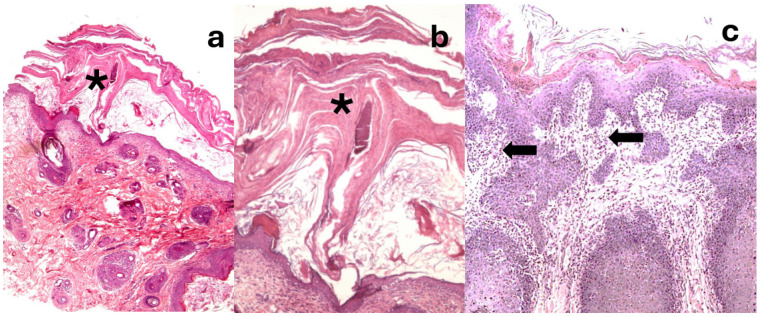
(**a**) Skin biopsy of dog case 9 (Hematoxylin/eosin). Irregular epidermal hyperplasia with severe parakeratotic hyperkeratosis (asterisk). (**b**) Case 9. Closer image of the severe parakeratotic hyperkeratosis (asterisk). (**c**) Skin biopsy of dog case 15 (Hematoxylin/eosin). Marked irregular epidermal hyperplasia with parakeratosis, extending to the hair follicle infundibula. In the dermis, note the presence of a perivascular to interstitial inflammatory infiltrate composed of polynuclear neutrophils, lymphocytes, and plasma cells (black arrows).

Histopathologic examination, performed in nine dogs, revealed consistent and distinctive microscopic alterations that supported the diagnosis. The epidermis showed moderate to severe irregular hyperplasia with basal papillomatosis, absence of the stratum granulosum, and prominent parakeratotic yperkeratosis (Figure 2a,b). Multifocally, spongiosis with exocytosis of lymphocytes and neutrophilic polymorphonuclear cells, as well as occasional apoptotic keratinocytes, was observed. The lesions extended into the follicular infundibula. In the superficial dermis, a perivascular to interstitial inflammatory infiltrate composed predominantly of neutrophils, lymphocytes, and plasma cells was consistently present (Figure 2c). These histological features closely resembled those described in previous reports of diet-related dermatoses associated with nutritional deficiencies.

All dogs were transitioned to a premium, nutritionally balanced commercial diet. Fifteen of the 22 animals (68%) received prednisolone (1 mg/kg/day for seven days) to alleviate moderate to severe pruritus and potentially enhance the intestinal absorption of trace elements and minerals. Two dogs were treated with oclacitinib (Apoquel^®^, Zoetis; Buenos Aires, Argentina) at 0.5 mg/kg every 12 h for 15 days, and one dog received hydroxyzine (2.2 mg/kg every 12 h for seven days). Four dogs (18%) were also treated with systemic antimicrobials following cytologic identification of intracellular cocci within neutrophils.

Systemic clinical signs such as lethargy and reduced activity resolved within one week of dietary change. Cutaneous lesions progressively improved and completely resolved between 15 and 30 days after the introduction of the balanced diet. Although quantitative data were not collected, the clinicians observed that lesion resolution appeared to occur more rapidly in the dogs treated with prednisolone compared to those managed with diet alone.

## 4. Discussion

The case series presented in this study exhibits clinical and histopathological characteristics consistent with those originally described by Sousa and coworkers in 1988 under the term “generic food dermatosis” [6]. In that seminal report, the authors proposed a link between the consumption of low-quality commercial pet foods and the development of a distinctive dermatosis characterized by parakeratotic hyperkeratosis and marked epidermal changes. After the initial description, reports of similar cases became exceedingly rare, and the condition appeared to have largely disappeared from the usual spectrum of dermatological presentations encountered in small-animal practice. It was generally assumed that improvements in commercial pet food manufacturing and quality control had effectively eliminated this problem. In recent years, however, we have observed a resurgence of this dermatosis in our region (Buenos Aires, Argentina), coinciding with a noticeable increase in the availability and consumption of inexpensive, nutritionally imbalanced animal feeds. The growing frequency of such cases has prompted renewed interest in this entity, as more patients are being referred to dermatology services with severe, treatment-resistant skin lesions ultimately attributable to poor dietary quality. In most cases, diagnosis can be strongly suspected based on a detailed anamnesis. Owners frequently report the onset of dermatological signs following a recent switch to low-cost, generic, or locally produced diets of uncertain composition. These diets are often marketed as “complete and balanced” but lack adequate nutritional standards or quality assurance.

Despite this shared dietary background, no consistent demographic pattern could be identified among affected animals (Supplementary Material, Table S1). The condition affected dogs of various breeds, both sexes, and a broad age range. While it appeared to occur more frequently in young adult dogs (mean age: 31 months), several older dogs (>6 years) were also affected, suggesting that chronic exposure or cumulative nutritional deficiency may also play a role in disease development.

Clinically, affected dogs present with crusted, scaly, and often erythematous lesions of variable severity. The lesions typically begin on the lips, periocular regions, and mucocutaneous junctions, with progressive extension to the distal limbs and, in more advanced cases, to the trunk and other parts of the integument. The condition is frequently pruritic, and secondary bacterial or yeast infections may complicate the clinical picture. The distribution and character of the lesions make the differential diagnosis broad, encompassing zinc-responsive dermatosis, superficial necrolytic dermatitis, generalized demodicosis, superficial pyoderma, dermatophytosis, and autoimmune diseases such as pemphigus foliaceus [4,9]. Other diagnostic tests were not performed due to the owners’ lack of resources. Tests to rule out Leishmania were also not carried out because the study was conducted in Buenos Aires, Argentina where, to date, no cases of this disease have been reported locally. Careful clinical evaluation, supported by laboratory and histopathological findings, is therefore essential to reach a definitive diagnosis.

Histopathological examination consistently reveals a characteristic pattern that supports the clinical suspicion. The predominant changes include moderate to severe epidermal hyperplasia with basal papillomatosis, pronounced parakeratotic hyperkeratosis, and variable spongiosis. Exocytosis of lymphocytes and neutrophilic polymorphonuclear cells is frequently observed within the epidermis, reflecting an ongoing inflammatory response. These features, while not pathognomonic, are highly suggestive when correlated with the dietary history and clinical distribution of lesions [9,10].

Therapeutic response provides an additional and often decisive diagnostic clue. Most affected dogs exhibit a rapid and marked improvement within days to weeks after being switched to a high-quality, nutritionally balanced commercial diet. The use of oral glucocorticoids at anti-inflammatory doses may accelerate recovery in severe or pruritic cases but is not always necessary once appropriate nutritional correction is achieved. The favorable outcome with simple dietary management underscores the pivotal role of nutrition in maintaining cutaneous homeostasis and highlights the need for greater regulatory oversight of commercial pet food products [11,12].

The reappearance of “generic food dermatosis” in our region raises important questions about the quality control and formulation of certain animal feeds currently available in the market. It also emphasizes the importance of thorough dietary history-taking in the dermatological evaluation of dogs presenting with hyperkeratotic or crusted lesions. Continued surveillance and reporting of similar cases are warranted to better understand the epidemiology of this condition and to promote awareness among veterinarians and pet owners regarding the critical link between diet quality and skin health [13].

Although the cause of the disease seems to be clear, the pathogenesis remains partially unknown. The most obvious explanation is that the clinical picture is a consequence of a deficiency of trace elements and/or essential amino acids and/or fatty acids [14,15]. The most severe and characteristic histological lesions are seen in the epidermis (e.g., parakeratosis) and are similar to those seen in zinc-deficient animals [9,10,14]. It is therefore possible to speculate that zinc deficiency in the epidermis plays a central role in the pathogenesis of this entity. Sousa et al. [5] also pointed to zinc deficiency as the most likely cause of this entity.

Zinc is a trace element essential for multiple metabolic processes, many of which are directly related to skin and coat integrity. It acts as a cofactor for numerous metalloenzymes, including RNA and DNA polymerases, and is therefore crucial for the rapid cell turnover of the epidermis [14]. Zinc also plays a central role in fatty acid biosynthesis, vitamin A metabolism, and in the regulation of both the immune and inflammatory responses [15]. Some of zinc’s key physiological roles include the stabilization of cellular and lysosomal membranes, regulation of metalloenzyme activity, and modulation of immune cell functions. Zinc deficiency can impair the activity of neutrophils and macrophages, disrupt the complement system, and hinder the production of molecules and cells required for proper immunological recognition [16]. Approximately 20% of the body’s zinc is found in the skin, where it participates in keratinization. Zinc deficiency may cause a decrease in zinc-related lytic enzymes, disrupting keratinization and increasing the epidermal turnover rate. This results in a very thick stratum corneum with retained nuclei (parakeratosis) [4,16].

Clinical signs of zinc deficiency are most evident at the cutaneous level. In young animals, however, deficiency may also impair growth and systemic development. Typical manifestations include poor quality coat, alopecia, delayed wound healing, conjunctivitis, keratitis, and in prolonged cases, weight loss. Reduced appetite, often due to impaired taste and smell, as well as generalized lymphadenopathy, are also common findings, particularly in growing animals [3,4,13].

Intestinal zinc absorption can be reduced when the diet contains high amounts of calcium, iron, or copper, as these minerals compete for the same absorption sites. Furthermore, the phytate present in cereals forms complexes with zinc, limiting its availability. In the past, most cases of zinc-responsive dermatosis in dogs were linked to the consumption of low-quality dry foods, formulated primarily with cereals or soy [13,16,17].

In any case, it is difficult to explain why other dogs living together with the affected dogs and eating the same diet are not affected. Sousa and colleagues [6] attempted to reproduce the entity by administering a diet like that of the diseased dogs to two puppies and one adult dog. None of these 3 dogs developed a dermatological disease. It is therefore possible that genetic factors also play a role in the development of the disease. In any case, to better understand this entity, it would be necessary to start with an analysis of the composition of the diets associated with these cases, to detect deficiencies in essential nutrients.

Another interesting point to note is the good response to prednisolone, which could be due to its anti-inflammatory and antipruritic action, but also to the facilitating effect on the absorption of some minerals such as zinc among others. It is important to note that complete healing of the lesions occurs between the 15th and 30th day of treatment. Follow-up of the patients included in this study for more than 6 months indicated that there was no recurrence of the condition if the diet was maintained with balanced, good-quality food.

Although certain methodological limitations must be acknowledged, the overall clinical evidence remains compelling. In this study, it was not possible to measure the levels of proteins, minerals, and vitamins in the diet used, nor to assess these nutritional parameters in the patients. This lack of direct nutritional analyses limits the ability to establish a causal relationship between the diet’s composition and the dermatological manifestations observed.

Nevertheless, the clinical dermatological pattern, the dermogram findings, and the consistent association between the consumption of the commercial pet food and the cutaneous conditions were key elements supporting the diagnostic suspicions.

## 5. Conclusions

Although dermatoses associated with low-quality or nutritionally deficient diets have largely disappeared from the scientific literature and are often regarded as conditions of the past, the present case series demonstrates that they remain clinically relevant, at least in certain regions of the world. Such disorders should therefore continue to be included in the differential diagnosis of canine dermatological diseases, particularly in patients affected by socioeconomic limitations, suboptimal feeding practices, or restricted access to high-quality diets. Further studies incorporating detailed nutritional analyses of the implicated diets are warranted to better identify the specific deficiencies involved and to aid in the prevention of this disease.

## Figures and Tables

**Table 1:** Nutritional composition of the commercial diet.

Parameter	Value
1. Crude protein (minimal)	24%
2. Fat (minimal)	13%
3. Fibers (maximum)	6%
4. Minerals (maximum)	7%
5. Calcium (maximum)	1.1%
6. Phosphorus (maximum)	0.95%
7. Humidity (maximum)	10%
8. Energy	4120 Kcal/kg (on dry basis)

**Table 2:** Blood tests. Hemogram and biochemistry evaluation.

Case	PCV (%)	RBC (/m^3^)	Hemogl (g/dL)	WBCs (/mm^3^)	Platelets (/mm^3^)	Glycemia (mg/dL)	Urea (mg/dL)	Creatinine (mg/dL)	ALT (U/L)	AST (U/L)	ALKP (U/L)	Total Serum Proteins (g/dL)	Albumin (g/dL)
1	34	5.49 × 10^6^	11.8	1.58 × 10^4^	1.45 × 10^5^	96	36	0.53	40	57	558	5.8	1.9
2	45	6.38 × 10^6^	16.2	6.4 × 10^3^	3.48 × 10^5^	72	20	0.95	53	62	310	5.2	2.1
3	42	5.29 × 10^6^	14	7.1 × 10^3^	4.2 × 10^5^	68	28	0.6	99	23	238	6.4	3.6
4	41	6.05 × 10^6^	14	6.3 × 10^3^	2.61 × 10^5^	85	42	0.8	56	48	253	6.2	2.8
5	35	5.84 × 10^6^	12.2	1.694 × 10^4^	4.37 × 10^5^	110	50	0.9	70	50	150	7	3
6	40	5.92 × 10^6^	13.8	8.4 × 10^3^	2.5 × 10^5^	94	40	0.75	45	45	420	6.5	2.6
7	36	5.55 × 10^6^	12	7.2 × 10^3^	3.6 × 10^5^	78	56	0.56	66	55	360	5.1	2.2

## Data Availability

The original contributions presented in this study are included in the article/Supplementary Material. Further inquiries can be directed to the corresponding author.

## Supplementary Materials

The following supporting information can be downloaded at: https://www.mdpi.com/article/10.3390/vetsci13010106/s1, Table S1: Signalment of the 22 dogs with dermatosis associated with feeding 1 food (generic food).
