# Supplementary figures and images for: Dermatosis Associated with Feeding Low-Quality Food (Generic Food Dermatosis): A Case Series
Source: Vet Sci. 2026 Jan 21;13(1):106. doi: 10.3390/vetsci13010106 (PMC12846578; doi:10.3390/vetsci13010106)

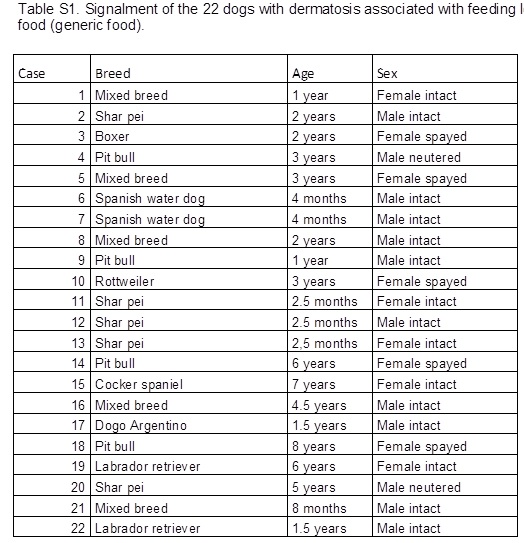

Supplement: Supplementary file 1 [file vetsci-13-00106-s001.zip › vetsci-4006796-supplementary.jpg]
